# Advancing the science behind human resources for health: highlights from the Health Policy and Systems Research Reader on Human Resources for Health

**DOI:** 10.1186/s12961-018-0346-5

**Published:** 2018-08-14

**Authors:** A.S. George, J. Campbell, A. Ghaffar, HPSR HRH reader collaborators, Seye Abimbola, Raeda AbuAlRub, Aarushi Bhatnagar, Marjolein Dieleman, Aku Kwamie, Veloshnee Govender, Luis Huicho, Uta Lehmann, Tim Martineau, Ligia Paina, N. S. Prashanth, Timothy Roberton, Krishna D. Rao, Kerry Scott, Veena Sriram, Stephanie Topp, Sophie Witter

**Affiliations:** 1 School of Public Health, University of the Western Cape, Private Bag x17, Bellville, Cape Town, 7535 South Africa; 2 Health Workforce, World Health Organization, 20 Avenue Appia, 1211 Geneva, Switzerland; 3 The Alliance for Health Policy and Systems Research, 20 Avenue Appia, 1211 Geneva, Switzerland

**Keywords:** Health policy and systems research, Human resources for health, Multi-disciplinarity, Rigour, Policy engagement

## Abstract

Health workers are central to people-centred health systems, resilient economies and sustainable development. Given the rising importance of the health workforce, changing human resource for health (HRH) policy and practice and recent health policy and systems research (HPSR) advances, it is critical to reassess and reinvigorate the science behind HRH as part of health systems strengthening and social development more broadly. Building on the recently published Health Policy and Systems Research Reader on Human Resources for Health (the Reader), this commentary reflects on the added value of HPSR underpinning HRH. HPSR does so by strengthening the multi-disciplinary base and rigour of HRH research by (1) valuing diverse research inferences and (2) deepening research enquiry and quality. It also anchors the relevance of HRH research for HRH policy and practice by (3) broadening conceptual boundaries and (4) strengthening policy engagement. Most importantly, HPSR enables us to transform HRH from being faceless numbers or units of health producers to the heart and soul of health systems and vital change agents in our communities and societies. Health workers’ identities and motivation, daily routines and negotiations, and training and working environments are at the centre of successes and failures of health interventions, health system functioning and broader social development. Further, in an increasingly complex globalised economy, the expansion of the health sector as an arena for employment and the liberalisation of labour markets has contributed to the unprecedented movement of health workers, many or most of whom are women, not only between public and private health sectors, but also across borders. Yet, these political, human development and labour market realities are often set aside or elided altogether. Health workers’ lives and livelihoods, their contributions and commitments, and their individual and collective agency are ignored. The science of HRH, offering new discoveries and deeper understanding of how universal health coverage and the Sustainable Development Goals are dependent on millions of health workers globally, has the potential to overcome this outdated and ineffective orthodoxy.

## Background

This Commentary is a joint publication by *Human Resources for Health* and *Health Research Policy and Systems*.

Health workers are central to people-centred health systems, resilient economies and sustainable development [1, 2]. Progress on these global goals depends on the effective deployment of capable and motivated health workers, in a timely manner to places where they are needed, so that they can provide a full range of high quality health services, respectfully and with accountability. The foundations for this affirmation of the strategic role of health workers were laid in the 2000s [3, 4]. Since then, human resources for health (HRH) policy and practice has evolved along with changing times.

While HRH policy previously focused on training, recruitment and deployment, recent concerns span issues related to migration, retention, dual practice, accountability, informal markets, gender bias and violence, as well as the need for HRH management and leadership in mixed and often poorly regulated health systems. Health policy and systems research (HPSR) gives us an opportunity to understand these contemporary shifts in HRH. HPSR seeks to understand and support how societies organise themselves in achieving collective health goals, and how different actors interact in the policy and implementation processes to contribute to policy outcomes (http://www.who.int/alliance-hpsr/about/hpsr/en/ Accessed 13 Feb 2018).

Given changing HRH policy and practice and recent HPSR advances, it is critical to reassess and reinvigorate the science behind HRH as part of health systems strengthening and social development more broadly. Building on the recently published Health Policy and Systems Research Reader on Human Resources for Health (the Reader) [5], this commentary reflects on the added value of HPSR underpinning HRH. HPSR does so by strengthening the multi-disciplinary base and rigour of HRH research by (1) valuing diverse research inferences and (2) deepening research enquiry and quality. It also anchors the relevance of HRH research for HRH policy and practice by (3) broadening conceptual boundaries and (4) strengthening policy engagement.

### Valuing diverse research inferences

**Fig. 1:**
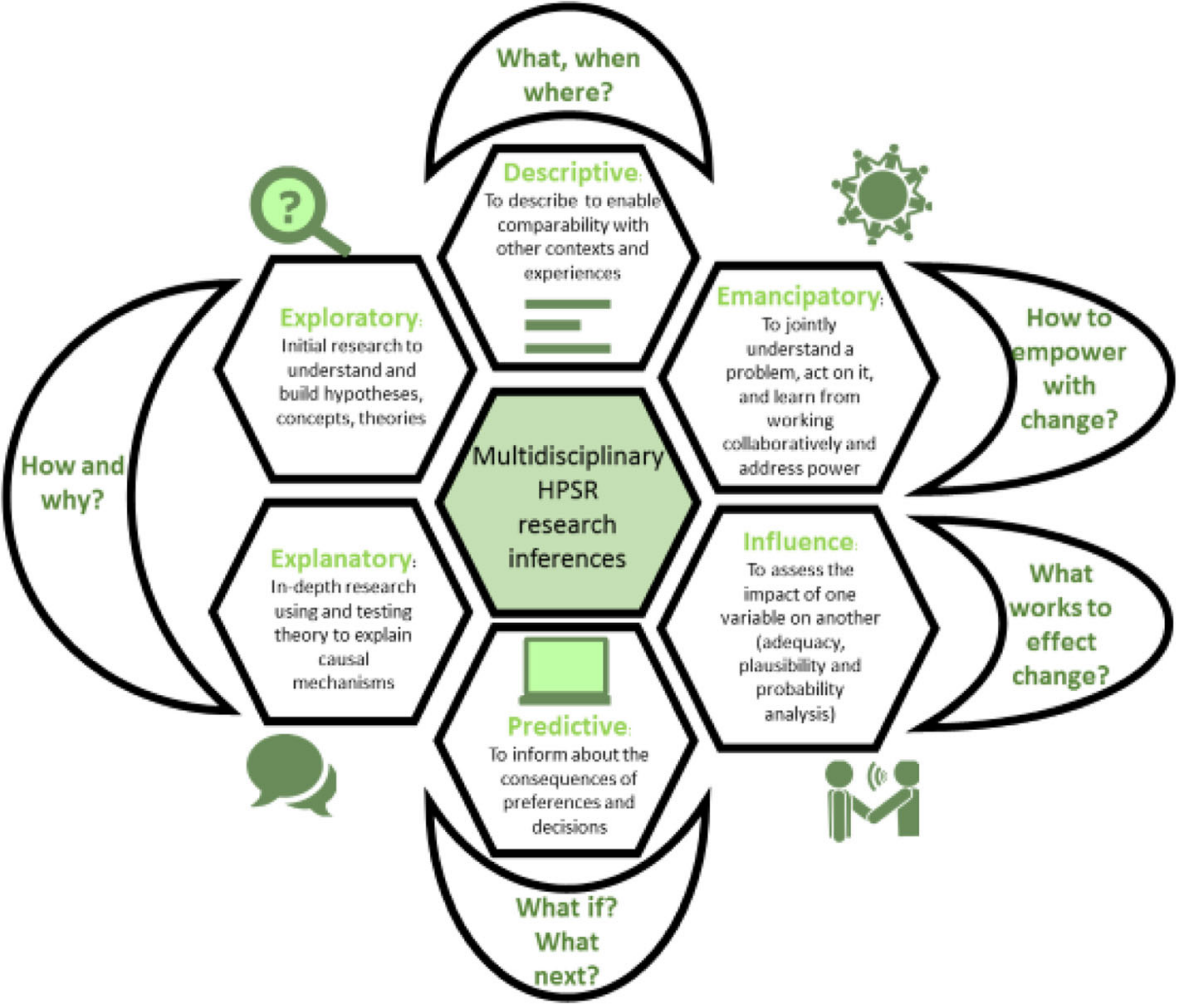
The mosaic of multi-disciplinary inferences in health policy and systems research

HPSR encourages a philosophy of science that is embedded, multi-disciplinary and multi-stakeholder in nature to ensure policy relevance and influence [6]. In contrast to the hierarchy of evidence that ranks study design by their ability to confirm attribution, HPSR argues for methodological fit dictated by the research question asked and its intended inference [7]. Accordingly, the Reader distinguishes between research that is descriptive, exploratory, explanatory, emancipatory, influence directed and predictive (Fig. 1).

Descriptive research serves a foundation for all research endeavours and provides the basis for contextualising research findings. Most of the HRH research reviewed for the Reader was descriptive in nature. From this large pool, the Reader sought to highlight efforts that used novel approaches or different data sources to better enumerate the distribution of health workers, whether in India [8] and Bangladesh [9] or across sub-Saharan Africa [10]. The Reader also selected descriptive research that systematically measured under-represented but critically important aspects of health workers’ lives such as workplace violence in Rwanda [11] and how health worker livelihoods depend on different sources of income in the Democratic Republic of the Congo [12]. Finally, the Reader also showcased how descriptive research helps to convey health worker insights on key performance mediators such as supervision in Zimbabwe [13] and organisational culture in Brazil [14], as well as their preferences for workplace location in Vietnam [15].

A key goal of the Reader is to encourage the science of HRH to move beyond its descriptive foundations and invest in other research inferences that also support evidence for HRH policy-making. For example, exploratory and explanatory HPSR seeks to understand underlying mechanisms, by focussing on ‘how’ and ‘why’ questions, using theories to guide and develop a deeper understanding of HRH.

Exploratory research is critical in uncovering the complexity inherent to health worker motivation [16], eliciting nuances in health workers’ perceptions of altruism [17] and organisational justice [18]. The Reader also showed how exploratory research can reveal the rationales for health worker decision-making related to dual practice [19] and migration [20]. It is also vital in developing new framing and conceptualisation of key social factors underpinning health worker performance [21], such as trust [22] and its abuse through health worker violence [23]. Finally, the Reader also demonstrated how exploratory research can illuminate the social processes that underpin organisational culture [24], such as the normalisation of corrupt practices and other detrimental coping mechanisms [25], as well as how transformative leadership and employee empowerment can be engines for change [26].

Building on the initial theories or conceptual understanding drawn from exploratory studies, explanatory research seeks to further test and advance theories in HRH. The Reader highlights explanatory research on the job preferences for rural deployment across various types of health workers in Peru [27] and the decision-space that supports district managers in Ghana [28]. Such research is critical in understanding why training and supervision initiatives work or fail [29]. For instance, the Reader includes explanatory research that unpacks why health workers reject innovations in health information systems [30], the contextual determinants of capacity-building efforts for district managers in India [31], and supervision in Malawi and Tanzania [32]. Explanatory research in the Reader also assesses how health workers negotiate posting and transfer systems in India [33] or pay for performance initiatives in Pakistan [34].

While doing research to understand how and why change occurs, HPSR can also guide change collaboratively through emancipatory approaches. Participatory action research [35] is an under-utilised research strategy in HPSR, but one that is highly valuable as it aims to empower participants in analysing, reflecting and acting upon their context (i.e. co-producing), thereby potentially transforming it. It inherently also shifts the power relations that conventionally structure research. The Reader includes research articles that reflect on these power dynamics and the meaning of co-producing research in learning sites with district managers in South Africa [36], as well as how it better enables understanding of resilience among managers in Kenya [37] and supervision in Zimbabwe [13]. The Reader also highlights innovative examples of how to use participatory research methods with health workers such as the use of life histories in Uganda [38] and concept mapping in Guatemala [39]. Finally, Reader sections also highlight how collaborative approaches with health workers are key to supporting performance, whether through better role definition in Egypt [40] or improved problem-solving teamwork [41] that supports quality improvement over time [42].

A key question for policy-makers is whether interventions or reforms work or have had intended or unintended effects; these make up the bulk of ex-post evaluations that aim to test the adequacy, plausibility and probability of influence. The Reader highlights innovative approaches to measuring effects of interventions on health workers, including the work environment on the responsiveness of health workers in Papua New Guinea [43], the effects of professionalism in Tanzania [44] and supervision in Ghana [45]. Examples of evaluating the impact of reforms such as Integrated Childhood Management of Illness in Benin [46] and Performance-Based Financing in Zambia [47] are also included. The Reader also considers macro-level impacts, such as the influence of global funding flows on health worker distribution, by contrasting experiences in Malawi and Zambia [48].

HPSR is also about informing stakeholders about the ex-ante implications of policy decisions, and is therefore predictive through scenario building, which can involve stakeholder participation and computer modelling. Rather than highlighting the multiple examples of workforce modelling that exist in HRH research, the Reader purposefully selected examples of workforce modelling that engaged policy stakeholders in the process of ex-ante assessment, whether in Australia [49] or in Guinea [50]. An important methodology for policy-makers is cost-effectiveness analysis, which can also be predictive in nature. Cost-effectiveness studies for ensuring retention in South Africa [51] or in Malawi [52] or for supporting community-based cadres in Ethiopia, Kenya and Indonesia [53] are an emerging field of evidence of vital importance.

These diverse HPSR inferences are not mutually exclusive. They can combine and accommodate diverse study designs and methods, each with their own principles of research quality. They are valuable in demonstrating how different types of research can answer the variety of enquiries needed to contribute in complementary ways to the breadth of understanding needed to inform HRH policy and practice.

### Deepening research enquiry and quality

Given the skewed nature of global research funding and capacity against low- and middle-income countries, the Reader particularly focussed on such contexts to contribute to efforts to redress this bias. Despite our call for contributions in all languages and multiple efforts to search for literature comprehensively, the Reader found few high-quality HPSR articles on HRH from Central Europe and Asia, the Middle East or from Latin America and the Caribbean. Substantial investments are required to strengthen HPSR on HRH in neglected linguistic and geographic regions, as well as in the collaborative HPSR networks that can sustain HRH research across linguistic and geographic regions.

With regards to institutional base, almost half of the selected articles in the Reader are exclusively dedicated to better understanding and supporting public sector health workers. While no research article in the Reader exclusively focussed on private sector health workers, several included and compared private health workers to public sector workers in their research [8, 9, 11, 22, 26, 43, 44, 49, 54, 55]. The Reader also recognised the porous boundaries between public and private through, for example, dual practice [15, 19]. While research supporting public sector health workers as the backbone of health systems is of vital importance, further comparative or stand-alone research with the private sector is also warranted.

As is common across HRH research, the kinds of health workers analysed in these articles were not always reported consistently or in a way that facilitated comparative analysis. Improved reporting against the International Labour Organisation’s international standard classification of occupations and on health worker type and gender, health system level, institutional base (public/private) and geographic location is vital to contextualise research and enable more appropriate generalisation for decision-making. National databases that routinely track the availability and distribution of health workers need investment to improve their quality, so that they can be more agile in capturing and tracking the nuanced and dynamic nature of an increasingly mobile and globalised health workforce [56]. This will also enable countries to provide national, public good for labour research and fulfil reporting requirements through the submission of National Health Workforce Accounts to WHO’s Global Health Observatory.

Apart from improving the quality of HRH data sources, the Reader also stressed further use and development of a broad range of research methods. Featured HRH research methods included experiments involving discrete choices [15, 57] or dictatorship games [17], time-use studies [13, 45], Likert scales and other types of scale development for measuring latent concepts such as motivation and job satisfaction [22, 26, 58], and vignettes to measure health worker performance [44]. While including examples of these known HRH research methods, the Reader highlights the need for improvement in how they are utilised to understand HRH. In addition, the Reader also highlights a range of social science methodologies as central to HRH research, including numerous examples of ethnography [18, 23, 25, 30, 58, 59], case study research [55, 60] and historical analysis [28, 61]. Innovations drawn from HPSR and applied to HRH showcased by the Reader include social network analysis [62], realist evaluation [31], action research [36, 42] and sampling through social media [20].

Despite showcasing such strong contributions of how HPSR strengthens HRH, the Reader also signals numerous areas for improving the quality of HRH research. Notwithstanding the emergence of quality checklists for various study designs, for example, we found research methods to be inconsistently reported across study designs. With some notable exceptions [63], researchers were also rarely reflexive about their own positionality and how it shaped the research process, participant responses and findings.

### Broadening conceptual boundaries to reflect health worker lived realities

At the core of HRH research and policy-making is a need to understand and potentially broaden the boundaries that define who counts as a health worker. The Reader calls to attention the importance of exploratory and explanatory research that examines where the boundaries are drawn, by whom and with what implications for the health workers involved, as well as research efforts that seek to descriptively count health workers in a more inclusive manner [64].

For example, in mapping the range of human resources that contribute to health, the Reader illustrates innovative research on doctors [10, 15, 19, 33, 61] and nurses [13, 17, 23, 55], but also highlights research on other kinds of health workers. Several studies focus on non-physician clinicians, whether exclusively [58] or alongside other health workers [26, 44]. Numerous articles also give voice to healthcare managers leading to greater understanding of their co-production of knowledge in South Africa [36], the historical evolution of their decision space in Ghana [28], their resilience under devolution in Kenya [37] and the contextual factors that support their capacity-building in India [31].

With a keen eye on community level providers, the Reader reveals health worker worldviews on their constrained livelihoods and lived experience amid sustained poverty and hunger in Ethiopia [59]. It reveals the community embeddedness of midwives in Mali [62] and of rural health workers in Papua New Guinea [54], alongside other organisational factors that impact on community cadre performance in Ghana [45], Guatemala [39] and Papua New Guinea [43]. While informal providers are often discounted, they are included in the Reader through efforts to enumerate the total workforce in India [8] and in Bangladesh [9].

A key contribution of HPSR is how it conceptualises important aspects of social relations that may otherwise be hard to recognise, measure and address [56]. For instance, a key social relation, often neglected in HRH due partially to the lack of sex-disaggregated data, is gender [65, 66]. The Reader highlights how gender bias filters into the framing of global policy on caregivers [67], prioritisation of nursing law in Lebanon [55], and the lived experience and family roles negotiated by caregivers in Ethiopia [59] and community cadres in Papua New Guinea [54]. Gender discrimination also underpins workplace violence in Rwanda [11], income levels in the Democratic Republic of the Congo [12] and opportunities for promotion in Uganda [38]. Efforts to recognise and address gender bias in the Reader include transformative training initiatives such as Health Workers for Change [41]. The Reader also noted certain gaps in research on gender dynamics in HRH. For example, while research in high-income countries is addressing gender and leadership in the health sector [68], no comparable research was found in low- and middle-income country contexts.

### Strengthening policy engagement

HPSR emphasises actor-oriented analysis, highlighting how health workers can be creative and dynamic agents working alongside patients, community members, managers and policy-makers to negotiate the diverse political interests and changing power relations that underpin health system complexities. A holistic understanding of health workers is critical in repositioning HPSR as key to strengthening HRH policy engagement by valuing stakeholder participation in research and by understanding the political nature of stakeholder interests and power in broader policy engagement.

HPSR directly elicits participation from key stakeholders through research that is emancipatory in nature (more detail below). Even if not directly collaborating with health workers and managers in the process of setting the research questions, undertaking the research or analysis, HPSR values engagement with decision-makers and other stakeholders as a means to strengthen research rigour and relevance. The Reader illustrates how stakeholder workshops were critical in validating research findings when understanding HRH policy-making in Lebanon [55] and Sierra Leone [69]. It also enabled HPSR to support policy deliberations, whether related to workforce planning in Australia [49] or Guinea [50] or in responding to sensitive issues such as workplace violence and gender discrimination in Rwanda [11].

HPSR also enables critical understanding of how HRH policies are negotiated and brokered among various stakeholders and their political interests [70]. The Reader highlights explanatory research about the policy processes that shape the roles, power and influence of doctors as a profession in Mexico [61], nurses in Lebanon [55] and caregivers at a global level [67]. Policy analysis can also explain what drives coherence between various aspects of HRH and maternal and child health policy [60] and the political economy driving HRH policy in post-conflict contexts such as in Sierra Leone [69]. In doing so, HPSR does not just work alongside HRH stakeholders, but ideally also balances autonomy and empathy to forge common ground among the diverse stakeholders and sectors involved in HRH decision-making.

## Conclusion

The Reader emerged from the desire to provide guidance on and examples of innovative HRH research, embracing health workers as creative and dynamic agents working alongside patients, community members, managers and policy-makers to address contemporary health system complexities. In doing so, the Reader promotes greater understanding and appreciation of the varied HPSR approaches that can be applied to HRH and provides resources that can be used for teaching and capacity development on HRH for researchers and practitioners alike. The highlighted HPSR articles attest that HPSR is catalytic to, and plays a vital and added value role in, advancing the science underpinning HRH. It does so by spurring disciplinary breadth and innovation that is vital for all fields of science while being anchored by an ethos of policy engagement. The combination deepens our understanding of the conceptual theories, lived experiences and pragmatic decisions that characterise the social relations, agency and interests of the diverse stakeholders and sectors that make up HRH.

Most importantly, HPSR enables us to transform HRH from being faceless numbers or units of health producers to the heart and soul of health systems and vital change agents in our communities and societies. Health workers’ identities and motivation, daily routines and negotiations, and training and working environments are at the centre of successes and failures of health interventions, health system functioning and broader social development. Further, in an increasingly complex globalised economy, the expansion of the health sector as an arena for employment and the liberalisation of labour markets has contributed to the unprecedented movement of health workers, many or most of whom are women, not only between public and private health sectors, but also across borders. How governments address these gender dynamics and broader socioeconomic outcomes is most evident in how they recognise and reward health workers. Yet, these political, human development and labour market realities are often set aside or elided altogether. Historic and orthodox conceptualisations – dominated by labels of ‘manpower planning’, ‘brain drain’, ‘task-shifting’ and ‘crisis’ – have perpetuated models where national and global decision-makers uniformly portray health workers as a function or cost of achieving health targets, health outcomes and, most recently, universal health coverage. Health workers’ lives and livelihoods, their contributions and commitments, and their individual and collective agency are ignored. The science of HRH, offering new discoveries and deeper understanding of how universal health coverage and the Sustainable Development Goals are dependent on millions of health workers globally, has the potential to overcome this outdated and ineffective orthodoxy.

## Abbreviations

HPSR: Health policy and systems research
HRH: Human resources for health
